# A Case of Intrathoracic Gastric Duplication Cyst Detected on Prenatal Ultrasound Examination

**DOI:** 10.1155/2018/5346920

**Published:** 2018-09-13

**Authors:** Hisako Yagi, Yoshino Kinjyo, Yukiko Chinen, Hayase Nitta, Tadatsugu Kinjo, Keiko Mekaru, Hitoshi Masamoto, Hideki Goya, Tomohide Yoshida, Naoya Sanabe, Yoichi Aoki

**Affiliations:** ^1^Department of Obstetrics and Gynecology, Graduate School of Medicine, University of the Ryukyus, 207 Uehara, Nishihara, Okinawa 903-0215, Japan; ^2^Department of Pediatrics, Graduate School of Medicine, University of the Ryukyus, 207 Uehara, Nishihara, Okinawa 903-0215, Japan; ^3^Department of Digestive and General Surgery, Graduate School of Medicine, University of the Ryukyus, 207 Uehara, Nishihara, Okinawa 903-0215, Japan

## Abstract

A 37-year-old (G4P3) woman was referred to our hospital at 32 weeks of gestation for the evaluation of a fetus with an intrathoracic cystic lesion. Ultrasonography and magnetic resonance imaging revealed that a fetal cystic lesion without a mucosal layer was located in the posterior mediastinum. These findings were consistent with a bronchogenic cyst. At 38 3/7 weeks of gestation, an elective cesarean section was performed because of her previous cesarean section. A female neonate without any external anomalies, weighing 2,442 g, with Apgar scores of 8 and 9, and requiring no resuscitation was born. Four weeks after delivery, the neonate was admitted because of respiratory distress due to mass effect. At right lateral thoracotomy, a 105 × 65 mm of solitary smooth-walled cyst containing serosanguineous fluid was found in the posterior mediastinum, which was excised completely. Histologic examination revealed the diagnosis of the mediastinal gastric duplication cyst. The neonate made an uneventful recovery. Accurate diagnosis is not necessary, but detection and continuous observation are logical. Although gastric duplication, particularly intrathoracic, is a rare pathology, it should be considered in the differential diagnosis of any intrathoracic cyst.

## 1. Introduction

Foregut duplication cysts are rare congenital anomalies of enteric origin; they constitute 10%–18% of all mediastinal lesions. They are further subdivided into bronchogenic, esophageal, gastric, enteric, and pancreatic cysts [1]. Gastric duplications account for less than 4% of all enteric duplications and most of them are located in the abdomen [2]. Although prenatal or early neonatal diagnosis is very important to avoid a complicated course, it is sometimes difficult. We report a case of an intrathoracic gastric duplication cyst detected on a prenatal ultrasound (US) examination.

## 2. Case Presentation

**Figure 1:**
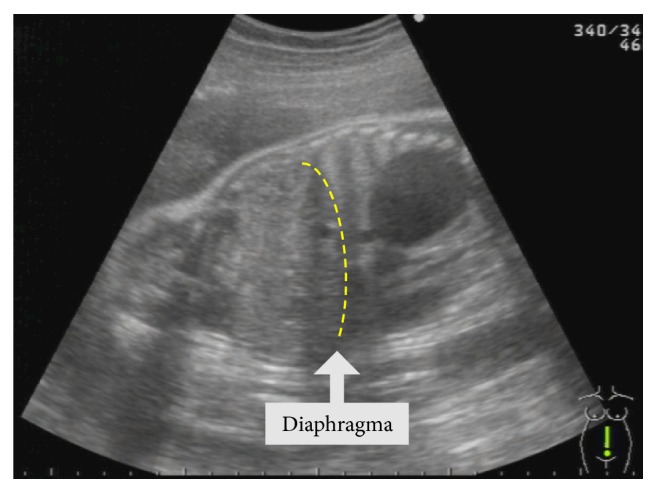
Ultrasound examination shows a 39 × 30 × 44-mm sized monocystic lesion in the mediastinum, in which the aortic arch was displaced upward.

**Figure 2:**
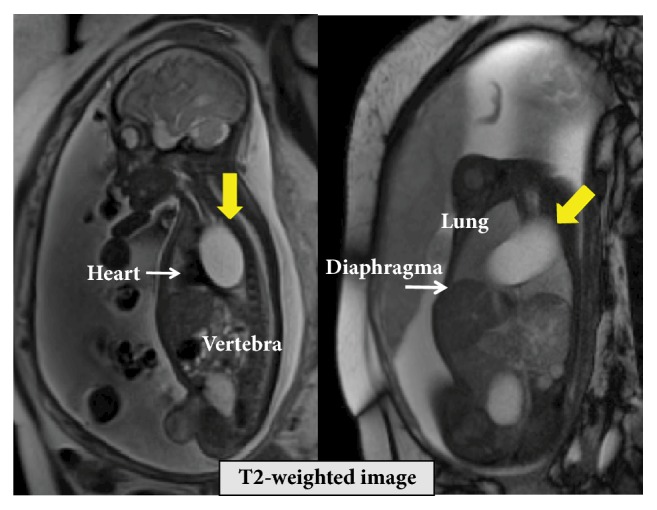
T2-weighted magnetic resonance imaging shows fetal cystic lesion located in the posterior mediastinum without communication to surrounding organs.

**Figure 3:**
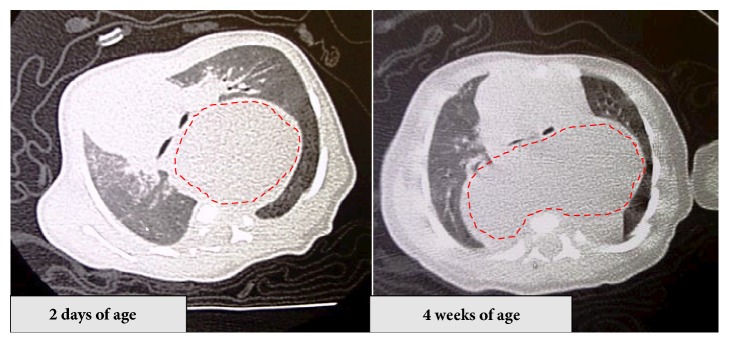
Four weeks after delivery, the neonate was admitted because of respiratory distress due to mass effect. Computed tomography scans (right panel: age 2 days, left panel: age 4 weeks) show enlargement of the mediastinal cystic lesion.

**Figure 4:**
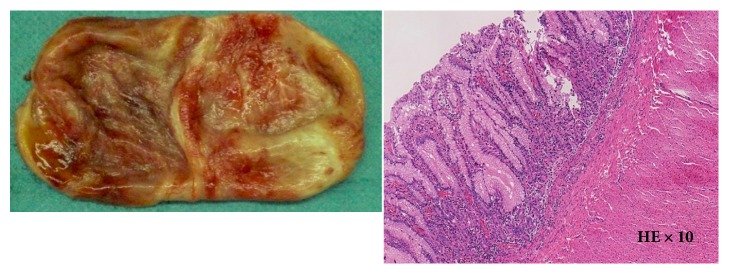
A 105 × 65 mm of solitary smooth-walled cyst containing serosanguineous fluid was excised completely (right panel). Histologic examination shows an inner lining of gastric mucosa and an outer smooth muscle coat (left panel), leading to the diagnosis of the mediastinal gastric duplication cyst.

A 37-year-old (G4P3) woman was referred to the University of the Ryukyus Hospital at 32 weeks of gestation for the evaluation of a fetus with an intrathoracic cystic lesion. An US examination revealed a 39 × 30 × 44-mm sized monocystic lesion in the mediastinum, in which the aortic arch was displaced upward (Figure 1). Magnetic resonance imaging (MRI) revealed that a fetal cystic lesion was located in the posterior mediastinum without communication to surrounding organs (Figure 2). A mucosal layer in the cyst could not be depicted by US and MRI; these findings were consistent with a bronchogenic cyst. Thereafter, her pregnancy course was uneventful. At 38 3/7 weeks of gestation, an elective cesarean section was performed because of her previous cesarean section. A female neonate without any external anomalies, weighing 2,442 g, with Apgar scores of 8 and 9, and requiring no resuscitation was born. Computed tomography (CT) scan revealed a monocystic lesion in the posterior mediastinum consistent with a bronchogenic cyst. Four weeks after delivery, the neonate was admitted to the pediatric surgery ward because of respiratory distress due to mass effect. CT scan revealed enlargement of the mediastinal cystic lesion (Figure 3), and surgery was performed. At right lateral thoracotomy, a 105 × 65 mm of solitary smooth-walled cyst containing serosanguineous fluid was found in the posterior mediastinum, which was excised completely. Histologic examination revealed an inner lining of gastric mucosa and an outer smooth muscle coat (Figure 4), leading to the diagnosis of the mediastinal gastric duplication cyst. The neonate made an uneventful recovery and was discharged on the seventh postoperative day.

## 3. Discussion

The diagnosis of intrathoracic cysts is challenging because of several possible pathologic findings, including bronchogenic cysts, neurenteric cysts, and other foregut duplication cysts. Most intrathoracic alimentary tract duplications present before the age of 2 years [3, 4]. Therefore, detecting the cyst prenatally is important, thereby leading to appropriate management at birth. Nakazawa et al. observed a mucosal layer in the cyst by postnatal CT scan, leading to the diagnosis of foregut duplication cyst [5]. In our patient, intrathoracic cyst was detected prenatally, and the cyst was monochamber and had smooth thin wall. We diagnosed it as bronchogenic cyst and not gastric duplication cyst. An accurate prenatal diagnosis seems difficult. Moreover, indications for antenatal intervention are limited, other than existence of hydrops fetalis [6]. Accurate diagnosis is not necessary, but detection and continuous observation are logical.

Duplication cysts can present various symptoms, such as dyspnea, stridor, or persistent cough, according to the location and type of the cysts. Asymptomatic patients later become symptomatic because of cyst enlargement, which was seen in our patient [1]. However, choosing between conservative treatment and resection for asymptomatic patients is controversial [7]. In our case, we were concerned about the potential complications of cyst removal in early neonatal period. However, serious mediastinitis due to cyst infection, malignant transformation [8], life threatening hematemesis or hemoptysis, and risk of bleeding from mucosal erosion [9] were reported. Early cyst removal should also be considered.

The etiology of enteric duplication remains speculative. The most accepted theory “split notochord syndrome” postulated the abnormal separation of the notochord from the endoderm, leading to enteric duplications [10]. Although gastric duplication, particularly intrathoracic, is a rare pathology [11, 12], it should be considered in the differential diagnosis of any intrathoracic cyst.
